# Outcomes of Hormone-Receptor Positive, HER2-Negative Breast Cancers by Race and Tumor Biological Features

**DOI:** 10.1093/jncics/pkaa072

**Published:** 2020-09-23

**Authors:** Halei C Benefield, Katherine E Reeder-Hayes, Hazel B Nichols, Benjamin C Calhoun, Michael I Love, Erin L Kirk, Joseph Geradts, Katherine A Hoadley, Stephen R Cole, H Shelton Earp, Andrew F Olshan, Lisa A Carey, Charles M Perou, Melissa A Troester

**Affiliations:** 1 Department of Epidemiology, Gillings School of Global Public Health, University of North Carolina at Chapel Hill, Chapel Hill, NC, USA; 2 Department of Medical Oncology, UNC Lineberger Comprehensive Cancer Center, University of North Carolina at Chapel Hill, Chapel Hill, NC, USA; 3 Department of Pathology and Laboratory Medicine, UNC Lineberger Comprehensive Cancer Center, University of North Carolina at Chapel Hill, Chapel Hill, NC, USA; 4 Department of Biostatistics, Gillings School of Global Public Health, University of North Carolina at Chapel Hill, Chapel Hill, NC, USA; 5 Department of Genetics, University of North Carolina at Chapel Hill, Chapel Hill, NC, USA; 6 Department of Pathology and Laboratory Medicine, East Carolina University, Greenville, NC, USA; 7 Department of Medicine and Pharmacology, University of North Carolina at Chapel Hill, Chapel Hill, NC, USA

## Abstract

**Background:**

Black women have higher hormone receptor positive (HR+) breast cancer mortality than White women. Early recurrence rates differ by race, but little is known about genomic predictors of early recurrence among HR+ women.

**Methods:**

Using data from the Carolina Breast Cancer Study (phase III, 2008-2013), we estimated associations between race and recurrence among nonmetastatic HR+/HER2-negative tumors, overall and by PAM50 Risk of Recurrence score, PAM50 intrinsic subtype, and tumor grade using survival curves and Cox models standardized for age and stage. Relative frequency differences (RFD) were estimated using multivariable linear regression. To assess intervention opportunities, we evaluated treatment patterns by race among patients with high-risk disease.

**Results:**

Black women had higher recurrence risk relative to White women (crude hazard ratio = 1.81, 95% confidence interval [CI] = 1.34 to 2.46), which remained elevated after standardizing for clinical covariates (hazard ratio = 1.42, 95% CI = 1.05 to 1.93). Racial disparities were most pronounced among those with high PAM50 Risk of Recurrence score (5-year standardized recurrence risk = 18.9%, 95% CI = 8.6% to 29.1% in Black women vs 12.5%, 95% CI = 2.0% to 23.0% in White women) and high grade (5-year standardized recurrence risk = 16.6%, 95% CI = 11.7% to 21.5% in Black women vs 12.0%, 95% CI = 7.3% to 16.7% in White women). However, Black women with high-grade tumors were statistically significantly less likely to initiate endocrine therapy (RFD = −8.3%, 95% CI = −15.9% to −0.6%) and experienced treatment delay more often than White women (RFD = +9.0%, 95% CI = 0.3% to 17.8%).

**Conclusions:**

Differences in recurrence by race appear greatest among women with aggressive tumors and may be influenced by treatment differences. Efforts to identify causes of variation in cancer treatment are critical to reducing outcome disparities.

Racial disparities in breast cancer outcomes have been described for decades and persist despite improvements in survival among all women (1). Based on 2018 Surveillance, Epidemiology, and End Results data, Black women were estimated to have a 41% higher breast cancer mortality rate compared with White women (2). Racial disparities are particularly pronounced among the clinically favorable hormone receptor-positive (HR+)/HER2 negative (HER2−) subtype (3–6). In a clinical trial where women with similar baseline states of health received the same standard of care, Black women with estrogen receptor (ER) positive/HER2− tumors had 5-year hazard of recurrence or death 1.58 times (95% CI = 1.19 to 2.10) that of White women (7).

Several studies suggest tumor biological differences may explain these outcome disparities. A recent report from the Trial Assigning Individualized Options for Treatment found that despite comparable treatment regimens and similar 21-gene assay recurrence scores, Black women with HR+ breast cancer had worse disease-free survival than White women (8). In the Carolina Breast Cancer Study (CBCS) phases I and II (1993-2001), we found that among HR+/HER2− patients, Black women had poorer breast cancer-specific and overall survival and, in phase III (2008-2013), were more likely to have aggressive intrinsic subtype (Luminal B, HER2-Enriched, and Basal-like), high-PAM50 assay risk of recurrence (ROR-PT) scores, and high tumor grade (3,9). However, CBCS phases I and II did not have recurrence data, and other data sources evaluating recurrence outcomes include relatively few Black participants. Thus, while several biological explanations for disparities are plausible, few studies have had sufficient data on intrinsic subtype, risk of recurrence scores, and recurrence outcomes in Black and White women.

Given the growing number of breast cancer survivors in the United States, which currently exceeds 3.5 million women, understanding recurrence is increasingly important (10). A large proportion of breast cancer deaths are attributable to recurrence after initial treatment for nonmetastatic disease (11,12). However, data collection for recurrence is resource intensive, and few population-based resources exist to study recurrence (13,14). The current analysis studies genomic predictors of recurrence using data from CBCS phase III, a population-based study of 3000 invasive cancer patients, comprising 50% Black and 50% White women. We estimate associations between race and recurrence, overall and by gene expression (PAM50 intrinsic subtype and ROR-PT score) and tumor grade. We also describe treatment differences between Black and White women with high-risk disease.

## Methods

### Study Population

**Figure 1.**
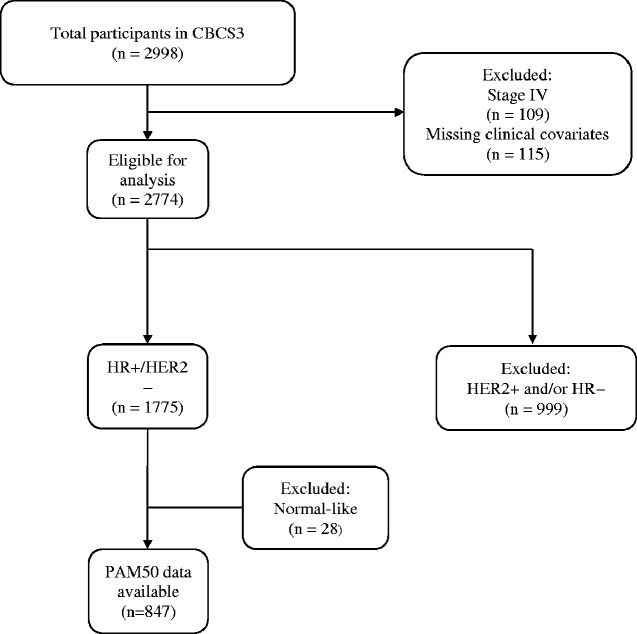
Flow diagram for study population. CBSC = Carolina Breast Cancer Study.

The CBCS is population-based study that began in North Carolina in 1993. Phase III of the study enrolled participants from 2008 to 2013; study details were described previously (15,16). Figure 1 depicts patient inclusion. Briefly, women aged 20-74 years diagnosed with first primary invasive breast cancer were enrolled using rapid case ascertainment. African American/Black and younger women (aged <50 years) were oversampled. Health history was collected during in-home interviews (17,18). Race was self-reported and categorized as White or African American/Black. Fewer than 2% of non-African American/non-Black participants self-identified as multiracial, Hispanic, or other races or ethnicities and were grouped with White race for statistical analyses. The study was approved by the Office of Human Research Ethics at the University of North Carolina at Chapel Hill (UNC). Informed consent was obtained from each participant.

### Clinical Characteristics

Tumor characteristics (size, node status, stage, grade, and ER and progesterone receptor [PR] status) and treatment data were abstracted from medical records and pathology reports. Stage IV tumors (n = 109) were excluded. Low or intermediate grade was defined as grade 1 or grade 2, and high grade was defined as grade 3. ER and PR status were defined by percent of cells staining positively using a 1% cutoff. HER2 status was derived from immunohistochemistry (IHC) and/or fluorescence in situ hybridization assay in pathology reports. Tumors that were ER-positive and/or PR-positive and HER2− were considered HR+/HER2− and included for analysis. Cases with missing clinical covariates (n = 115, 4%) were excluded.

### Genomic Assessment

The details of RNA isolation and quantification were published previously (9). Briefly, at UNC, RNA was isolated from formalin-fixed, paraffin-embedded tumor blocks using Qiagen RNeasy kit and quantified using NanoString assay (19). Quality control was performed using NanoString nSolver software. Data that passed quality control were normalized following nCounter protocol, including background subtraction, positive control normalization, and reference gene normalization. Normalized data were log2 transformed, standardized across samples, and median centered across genes. PAM50 predictor was used to categorize tumors as Luminal A, Luminal B, HER2-enriched, Basal-like, and normal-like and to calculate risk of recurrence score, an integrated score that incorporates subtype with additional weighting by proliferation gene signature and tumor size (ROR-PT) (9,20). The ROR-PT score predicts risk of distant recurrence in HR+ breast cancer (21,22). Normal-like (n = 28) were excluded from genomic analyses due to insufficient tumor cellularity. High ROR-PT was greater than 64.71, and low or intermediate ROR-PT was 64.71 or less per established protocol (20).

### Outcome Assessment

Time to recurrence was defined as time from diagnosis to subsequent recurrent breast cancer (local, regional, or distant). Breast cancer recurrence and date were verified using the medical records of women who reported having a recurrence during telephone follow-up, which occurred at 9, 18, 38, 66, 80, 92, and 104 months from enrollment. Recurrence was verified by reviewing the medical record for all available information, including managing physician’s notes, imaging studies, biopsy reports, and pathology reports. Abstraction for this analysis is complete through September 2018. Among 1775 eligible women, 170 recurrences were identified over a median follow-up of 6.68 years (range = 0.27-10.16). Women without a recurrence were censored at date of last contact. Ninety-two deaths of unknown cause without recurrence were identified by medical record and censored at date of death.

### Statistical Analyses

Linear binomial regression was used to calculate relative frequency differences (RFD), interpretable as the percentage difference between index and referent groups, and 95% confidence intervals (CI) as the measure of association between race and clinical covariates, adjusted for age at diagnosis. Differences in characteristics by race were also evaluated using the χ^2^ test. Crude hazard ratios were calculated using Cox proportional hazards models.

We used inverse probability of exposure weighting to adjust for baseline covariates in recurrence analyses (23,24). Inverse probability weighting accounts for baseline characteristics similar to adjusted Cox models, but does not force proportionality of hazards within all strata and allows standardized survival curve estimates to assess recurrence patterns over time. To assign weights to estimate the association of race with recurrence risk among all HR+/HER2−, we used logistic regression to calculate the probability of belonging to each group (Black vs White) accounting for age at diagnosis, grade (1, 2, or 3), node status (positive vs negative), and tumor size (≤2 cm vs >2 cm). To assign weights to estimate the association of race jointly stratified by tumor feature, we used multinomial logistic regression to calculate the probability of belonging to each group adjusting for age and stage. These probabilities were used to calculate stabilized inverse probability of exposure weights. The mean weight for all models was 1.0 (range = 0.23-3.00). Standardized hazard ratios were calculated using inverse probability weighted Cox proportional hazards models. The proportional hazards assumption was verified graphically and by Wald test of interaction term between group and survival time. Recurrence curves plots were terminated when the smallest subgroup had less than 10% remaining, but log-rank comparisons accounted for all recurrence events (25). Log-rank tests were adjusted for multiple comparisons using Bonferroni correction. Standardized recurrence curves were used to calculate 5-year risk of recurrence.

The CBCS was designed to examine the interaction of tumor biology and health-care access disparities. Although this analysis was focused on the comparison of outcomes within biological subgroups, we also explored treatment characteristics to identify potential targets for intervention among women with high-risk tumors. Treatment covariates were examined among grade 3 tumors, because grade is widely assessed and correlates strongly with high-risk molecular subgroups (18,20). Complete treatment data were available for 437 of 441 high grade participants (99%). We assessed chemotherapy receipt, endocrine therapy receipt, treatment delay (>30 days to first treatment of any modality), and health insurance status at interview. Differences were evaluated using χ^2^ test and RFDs adjusted for stage. We performed sensitivity analyses to assess treatment differences among high ROR-PT tumors and, separately, those with positive lymph nodes. We also assessed treatment differences among women with low and intermediate grade tumors.

Statistical analyses were conducted using SAS version 9.4 (SAS Institute Inc, Cary, NC). All tests were 2-sided and a *P* less than .05 was considered statistically significant.

## Results

### Overall

Among 1775 eligible women with HR+/HER2− stage I-III breast cancer, Black and White women exhibited statistically significantly different baseline clinical characteristics. Black women had high grade tumors nearly one-third (31.2%) of the time and were statistically significantly more likely to have high grade tumors than White women. Black women also had node positivity, large tumor size, and stage III more frequently than White women (Table 1).

**Table 1.** Characteristics of HR+/HER2− participants in the CBCS

Characteristic	White No. (%)	Black No. (%)	RFD, % (95% CI)^a^	*P* ^b^
All	766 (43)	1009 (57)	–	–
Age at diagnosis, y				
<50	483 (47.9)	358 (46.7)	0.0	
≥50	526 (52.1)	408 (53.3)	+1.1 (−3.6 to5.8)	.64
Menopausal status				
Premenopausal	443 (43.9)	333 (43.5)	0.0	
Postmenopausal	566 (56.1)	433 (57.5)	0.0 (−4.2 to 5.1)	.86
Combined grade				
1	327 (32.4)	178 (23.2)	0.0	
2	480 (47.6)	349 (45.6)	+6.4 (1.2 to 11.6)	
3	202 (20.0)	239 (31.2)	+17.3 (12.8 to 21.7)	<.001
Tumor size, cm				
≤2	657 (65.1)	408 (53.3)	0.0	
>2	352 (34.9)	358 (46.7)	+10.6 (6.0 to 15.1)	<.001
Node status				
Negative	682 (67.6)	466 (60.8)	0.0	
Positive	327 (32.4)	300 (39.2)	+ 5.5 (1.0 to 9.9)	.003
Stage				
I	547 (54.2)	325 (42.4)	0.0	
II	358 (35.5)	332 (43.3)	+10.0 (5.1 to 14.9)	
III	104 (10.3)	109 (14.2)	+6.5 (1.5 to 11.6)	<.001
Intrinsic subtype				
Luminal A	348 (73.7)	221 (59.1)	0.0	
Luminal B	83 (17.4)	94 (25.1)	+9.8 (3.5 to 16.0)	
HER2-Enriched	4 (0.9)	2 (0.5)	−0.2 (−2.6 to 2.2)	
Basal-like	38 (8.1)	57 (15.2)	+9.3 (3.7 to 14.9)	<.001
Missing	536	392		
ROR-PT score				
Low	149 (31.6)	73 (19.5)	0.0	
Medium	283 (60.0)	235 (62.8)	+10.7 (4.3 to 17.2)	
High	40 (8.5)	66 (17.7)	+23.1 (12.7 to 33.6)	<.001
Missing	537	392		

a RFD comparing Black women with White women, adjusted for age at diagnosis. CBCS = Carolina Breast Cancer Study; HR+, hormone receptor-positive; RFD = relative frequency difference; ROR-PT = PAM50 risk of recurrence score

b Two-sided χ^2^ test.

Black women with HR+/HER2− tumors had statistically significantly higher risk of recurrence relative to White women (crude HR = 1.81, 95% CI = 1.34 to 2.46), even after adjusting for age, grade, node status, and tumor size (standardized HR = 1.42, 95% CI = 1.05 to 1.93). To assess whether these differences were driven by genomic or histologic features, we performed stratified analysis by ROR-PT score, Luminal A vs B intrinsic subtype, and grade.

### ROR-PT High vs Medium or Low

**Figure 2.**
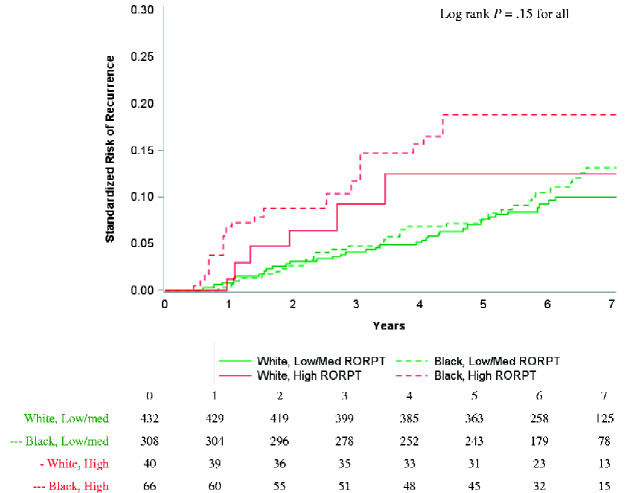
Standardized risk of recurrence among hormone receptor-positive/HER2− cases stratified by race and PAM50 risk of recurrence (ROR-PT) score. Pairwise log-rank tests were performed with Bonferroni correction for multiple comparisons. No pairwise comparisons were statistically significantly different. Risk was standardized for age and stage.

We compared recurrence risk by ROR-PT score among 846 women with genomic data available. We found that Black vs White recurrence curves (standardized for age and stage) separated early among women with high ROR-PT (Figure 2), with standardized 5-year recurrence risk of 18.9% (95% CI = 8.6% to 29.1%) for Black women vs 12.5% (95% CI = 2.0% to 23.0%) for White women (Table 2). There were no marked differences by race among women with low or medium ROR-PT (5-year recurrence risk of 7.6% for both races: 95% CI for Black women = 4.6% to 10.5%, White women = 5.1% to 10.2%).

**Table 2.** Standardized^a^ 5-year risk of recurrence, hazard ratios for recurrence risk, and 95% confidence intervals stratified by race and biological feature

Molecular subgroup and race	No. (No. of recurrences)	Crude 5-y risk of recurrence, % (95% CI)	Standardized^a^ 5-y risk of recurrence, % (95% CI)	Standardized^a^ HR (95% CI)	*P*
Low or medium ROR-PT					
White	432 (36)	6.6 (4.2 to9.0)	7.6 (5.1 to 10.2)	1.0	
Black	308 (34)	7.4 (4.4 to 10.3)	7.6 (4.6 to 10.5)	1.26 (0.80 to 1.97)	.37
High ROR-PT					
White	40 (6)	15.0 (3.9 to 26.1)	12.5 (2.0 to 23.0)	1.36 (0.52 to 3.56)	.53
Black	66 (16)	25.0 (14.3 to 35.6)	18.9 (8.6 to 29.1)	2.13 (1.07 to 4.26)	.03
Luminal A					
White	348 (25)	5.7 (3.2 to 8.2)	6.0 (3.4 to 8.6)	1.0	
Black	221 (22)	6.3 (3.0 to 9.6)	6.5 (3.1 to 9.9)	1.42 (0.81 to 2.47)	.22
Luminal B					
White	83 (9)	8.7 (2.5 to 14.8)	9.7 (3.3 to 16.2)	1.57 (0.75 to 3.30)	.23
Black	94 (17)	15.4 (8.0 to 22.8)	10.1 (3.9 to 16.3)	1.96 (1.02 to 3.80)	.04
Low or int grade					
White	807 (47)	4.5 (3.0 to 5.9)	5.3 (3.8 to 6.9)	1.0	
Black	527 (45)	5.9 (3.8 to 7.9)	6. (4.0 to 8.2)	1.34 (0.91 to 1.97)	.14
High grade					
White	202 (27)	11.8 (7.3 to 16.3)	12.0 (7.3 to 16.7)	2.14 (1.36 to 3.37)	.001
Black	239 (51)	20.8 (15.6 to 26.1)	16.6 (11.7 to 21.5)	2.80 (1.86 to 4.23)	<.001

a Standardized for age and stage. CI = confidence interval; HR = hazard ratio; int = intermediate; ROR-PT = risk of recurrence, proliferation and tumor size weighted.

### Luminal A vs B

**Figure 3.**
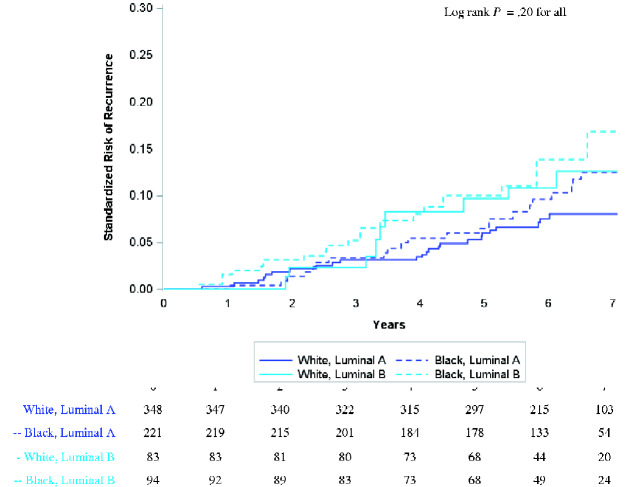
Standardized risk of recurrence among hormone receptor-positive/HER2− cases stratified by race and luminal subtype. Pairwise log-rank tests were performed with Bonferroni correction for multiple comparisons. No pairwise comparisons were statistically significantly different. Risk was standardized for age and stage.

We compared recurrence risk by luminal subtype among 747 women with tumors classified as Luminal A or B. Relative to ROR-PT classes, racial differences were attenuated within Luminal subtypes. Standardized survival curves for both Luminal A and Luminal B showed less separation by race (Figure 3). Black and White women with Luminal B breast cancer had comparable 5-year risks of recurrence of approximately 10% (Table 2).

### Grade High (Grade 3) vs Low or Intermediate (Grade 1 or 2)

**Figure 4.**
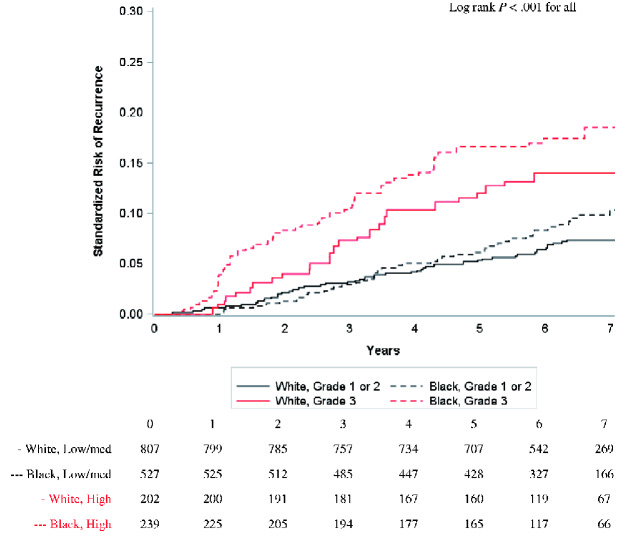
Standardized risk of recurrence among hormone receptor-positive/HER2− tumors stratified by race and grade. Pairwise log-rank tests were performed with Bonferroni correction for multiple comparisons. Compared with White women with low or intermediate grade, White women with high grade tumors (*P* < .001) and Black women with high grade tumors (*P* < .001) were statistically significantly different. Compared with Black women with low or intermediate grade tumors, Black women with high grade tumors were statistically significantly different (*P* = .005). No other pairwise comparisons were statistically significant. Risk was standardized for age and stage.

To evaluate racial differences within risk subgroups available in routine clinical practice, we also performed stratified analyses by overall tumor grade in the full study population (n = 1775). Grade is strongly associated with high-risk features and serves as a prognostic and predictive marker in the absence of gene profiling assays (18,20). We observed early separation of recurrence curves by race among women with high grade tumors (Figure 4). At 5 years, Black women with high grade tumors had a recurrence risk of 16.6% (95% CI = 11.7% to 21.5%), compared with 12.0% (95% CI = 7.3% to 16.7%) among White women. By comparison, Black and White women with low grade tumors had similar 5-year recurrence risk (Table 2).

Patterns of treatment may explain early recurrence risk among Black women, and therefore, we compared treatment characteristics by race among women with HR+/HER2−, high grade tumors. After adjusting for stage, Black women were less likely to initiate endocrine therapy (RFD = −8.3%, 95% CI = −15.9% to −0.6%) and more likely to experience treatment delay (>30 days to first treatment of any modality; RFD = +9.0%, 95% CI = 0.3% to 17.8%). Insurance status was not different by race (Table 3). Sensitivity analyses among tumors with a high ROR-PT score and among cases with positive lymph node status showed similar findings (data not shown). Among women with low or intermediate grade tumors, frequencies of initiation of chemotherapy (37% among White women, 41% among Black women; RFD = −0.2%, 95% CI = −3.6% to 3.1%), initiation of endocrine therapy (92% among White women, 91% among Black women; RFD = −1.6%, 95% CI = −4.6% to 1.4%), and treatment delay (34% among White women, 40% among Black women; RFD = +4.8%, 95% CI = −0.6% to 10.1%) were not statistically significantly different by race.

**Table 3.** RFD and 95% confidence intervals for treatment history and health insurance status for Black vs White women^a^

Treatment history and health insurance status	White, No. (%) n = 202	Black, No. (%) n = 235	*P* ^b^	RFD^c^, %(95% CI)
Chemotherapy				
Yes	155 (76.7)	204 (86.8)	.006	+0.1 (−7.1 to 7.3)
No	47 (23.3	31 (13.1)		0.0
Endocrine therapy				
Yes	170 (84.1)	178 (75.7)	.03	−8.3 (−15.9 to −0.6)
No	32 (15.8)	57 (24.3)		0.0
Treatment delay^d^				
Yes (>30 d)	52 (25.7)	85 (36.2)	.02	+9.0 (0.3 to 17.8)
No (<30 d)	150 (74.3)	150 (63.8)		0.0
Health insurance at interview				
No	15 (7.4)	23 (9.8)	.38	+6.0 (−0.9 to 13.0)
Yes	187 (92.6)	212 (90.2)		0.0

a Four participants had 1 or more missing treatment covariates. Analysis restricted to grade 3 hormone receptor-positive/HER2− tumors. RFD = relative frequency difference.

b Two-sided χ^2^ test.

c Adjusted for stage.

d Defined as more than 30 days to first treatment of any modality.

## Discussion

In a diverse, population-based cohort of incident HR+/HER2− cases, our study found substantial racial disparities in breast cancer recurrence, with Black women showing crude recurrence risk nearly twice that of White women. Overall, higher risk of recurrence among Black women was reduced but not eliminated after adjusting for baseline tumor characteristics. Disparities in recurrence patterns were most striking in high-risk subgroups of HR+/HER2− patients, notably those with a high ROR-PT score and high grade. These high-risk women may be most in need of appropriate treatment, and therefore treatment differences identified in Black vs White women (treatment delay and lower endocrine therapy initiation) are important targets to close these recurrence gaps.

Disparities in various survival outcomes by race among HR+/HER2− tumors have been consistently reported (3,5–8,26–28). An earlier analysis by O’Brien et al. (3) of IHC-based intrinsic subtypes in phases I and II (1993-2001) of CBCS found that Black women had higher breast cancer-specific mortality among Luminal A tumors (HR = 1.9, 95% CI = 1.3 to 2.8, standardized for age, stage, and date of diagnosis), defined by IHC markers as HR+/HER2−, but no other subtype. These findings did not address recurrence specifically, but overall the racial disparities observed are consistent with our results. The current analysis extends these earlier findings by examining standardized recurrence curves. In contrast to a single HR, which conveys relative risk of recurrence averaged over the study duration, standardized recurrence curves offer a visual representation of absolute risk of recurrence throughout follow-up, showing changes in outcome patterns over time. Examining recurrence in this manner revealed that disparities may be concentrated within high-risk strata, shown by the early, sustained separation of recurrence curves. Aggressive tumor biology among Black women has been hypothesized to explain disparities within HR+/HER2− disease. Indeed, we previously reported that among women in CBCS3 with clinically defined HR+/HER2− tumors, Black women were more likely to have aggressive non-Luminal A subtypes, particularly at younger ages (9). However, in our current analysis, stratifying by molecular subtype did not eliminate disparities in HR+/HER2− recurrence. Inability of genomic characteristics to fully account for disparities is consistent with other reports. Another study by Kroenke et al. (28) found that the association between race and recurrence remained statistically significant after adjusting for PAM50 intrinsic subtype (HR = 1.65, 95% CI = 1.06 to 2.57) but did not examine HR+/HER2− cases. Keenan et al. (29) similarly reported that adjusting for intrinsic subtype attenuated the association of race with recurrence, but with low precision (HR = 1.35, 95% CI = 0.62 to 2.95) and across all clinical subtypes. Our study includes a higher proportion of Black women [49% vs 8% in Kroenke et al. (28) and 14% in Keenan et al. (29)] and a substantial population of HR+/HER2− cancers, adding additional resolution to a growing body of evidence that tumor biology only partially accounts for breast cancer disparities among Black women.

Given the persistent disparities observed within high-risk HR+/HER2− women, it is important to identify plausible causes. There are a number of reasons why racial disparities might be concentrated among women with higher risk tumors. Because their tumors are high risk, these women may be more vulnerable to inadequate locoregional or systemic therapy. The standard of care for HR+/HER2− disease includes endocrine therapy, even for high-risk tumors, but in our study Black women with high grade HR+/HER2− tumors were less likely to initiate endocrine therapy and were more likely to experience delay in treatment initiation compared with White women (30). These differences were not observed among women with low or intermediate grade tumors. We also saw that a larger proportion of Black women with high grade tumors initiated chemotherapy compared with White women. This may be due to racial differences in uptake of the Oncotype DX clinical genomic assay, which informs the decision to use adjuvant chemotherapy in early-stage HR+/HER2− breast cancer (31,32). Unfortunately, racial disparities in treatment are well documented across the entire breast cancer care continuum, including initiation of endocrine therapy, treatment delay, surgery and radiation, and adherence (33–37). Thus, a more thorough analysis of treatment is needed to identify the most effective intervention strategies for reducing these inequities.

Strengths of this study include a large population-based cohort composed of nearly 50% Black women, extensive clinical and pathological data, detailed follow-up data, and RNA-based genomic data using a central laboratory. We were able to integrate multiple data types, ranging from tumor biology to treatment history, and our data show a convincing picture that even in the first several years following diagnosis, racial disparities are emerging due to the combined effects of different care and different rates of high-risk tumors.

Our findings should nonetheless be considered in light of some limitations. First, we considered endocrine therapy initiation, but not adherence, which warrants further consideration (34,38,39). We were missing adherence data on a large proportion of initiators, but we anticipate that adherence would be more impactful for late recurrence. Second, although our study is larger than previous studies of intrinsic subtype by race, recurrence rates are low in the early years following diagnosis (<2% per year on average), which limited our precision and precluded analysis of interactions between race and biological feature. Furthermore, early recurrence patterns may differ from longer term patterns. For example, our data did not show striking differences in recurrence when comparing low- and high-risk White women, but the predictors evaluated (ROR-PT and Luminal A and B) have consistently demonstrated effects on long-term survival and late recurrence in other studies (40,41). Nonetheless, disparities in the early window following diagnosis are important and have consequences for long-term survival. Moreover, they may be targetable by clinical strategies that increase rates of guideline-concordant endocrine initiation among Black women with HR+/HER2− tumors, especially among those with high-risk tumors. A final limitation of the current analysis is that commonly used genomic tests, such as the 21-gene recurrence assay, were available for few patients. However, the 21-gene assay and PAM50 tend to have relatively high concordance, so it will also be important to assess whether racial disparities are more pronounced among patients with Oncotype DX high-risk scores (21).

In summary, we show that the higher incidence of aggressive tumor subtypes, together with disparities in treatment, result in racial disparities in recurrence for HR+/HER2− breast cancer. Differences in initiation of endocrine therapy among higher risk women may play a role in driving these disparities. Continued research that integrates biological data with access-to-care measures is critical to reducing recurrence among Black women with high-risk HR+/HER2 tumors.

## Funding

This work was supported by a grant from the UNC Lineberger Comprehensive Cancer Center funded by the University Cancer Research Fund (LCCC2017T204), Susan G. Komen Foundation, the National Cancer Institute of the National Institutes of Health (P50-CA58223, U01-CA179715 to MAT and CMP, T32-CA057726 to HCB, F30-CA236199 to HCB), and the National Institute of Environmental Health Sciences of the National Institutes of Health (P30-ES010126 to MAT). HCB is a recipient of the Gertrude B. Elion Mentored Medical Student Research Award of Triangle Community Foundation.

## Notes

**Role of the funder:** This content is solely the responsibility of the authors and does not necessarily represent the official views of the National Institutes of Health. The funder had no role in study design, data collection, analysis or interpretation, or writing of the manuscript.

**Disclosures:** CMP is an equity stock holder, consultant, and Board of Director Member, of BioClassifier LLC and GeneCentric Diagnostics. CMP is also listed an inventor on patent applications on the Breast PAM50 assay. Authors have no other conflicts of interest to report.

**Prior presentation:** A portion of this manuscript was presented as a poster presentation at the AACR Conference on The Science of Cancer Health Disparities in Racial/Ethnic Minorities and the Medically Underserved September 20-23, 2019 in San Francisco, CA.

**Author contributions:** HCB, HBN, BCC, MIL, AFO, and MAT participated in conception and design of the work; ELK, AFO, CMP, and MAT participated in data collection; HCB, KRH, HBN, BCC, MIL, KAH, SRC, AFO, and MAT contributed to data analysis and/or interpretation; HCB drafted the article; all authors participated in critical review of the article and final approval of the version to be published.

## Data Availability

Data underlying this study may be requested at the Carolina Breast Cancer Study website found at the following link: https://unclineberger.org/cbcs/for-researchers/.
